# Belatacept in Solid Organ Transplantation: Current Kidney Applications, Future Perspectives in Other Organs, and Clinical Implications

**DOI:** 10.3390/ph19020196

**Published:** 2026-01-23

**Authors:** Salvatore Di Maria, Alessio Provenzani

**Affiliations:** 1 School of Specialization in Hospital Pharmacy, University of Catania, 95131 Catania, Italy; dimaria092s@gmail.com; 2 IRCCS ISMETT, 90127 Palermo, Italy; 3 UPMC Italy, 90133 Palermo, Italy

**Keywords:** belatacept, solid organ transplantation, immunosuppressive drugs, tacrolimus, cyclosporine

## Abstract

Belatacept, a selective costimulation blocker targeting the CD28–CD80/86 pathway, represents a major innovation in solid organ transplantation immunosuppression. By providing upstream inhibition of T-cell activation without calcineurin inhibition, belatacept offers the potential for improved long-term graft and patient outcomes with reduced nephrotoxicity and metabolic adverse effects. This review summarizes the mechanistic rationale, pivotal evidence, and clinical experience supporting the use of belatacept as first-line or conversion therapy in solid organ transplantation, while addressing safety, pharmacoeconomic impact, and future research directions. A comprehensive analysis of pivotal phase II–III trials (BENEFIT, BENEFIT-EXT), recent prospective conversion studies, and ongoing trials in liver, heart, and lung transplantation was performed. Safety data and health–economic evaluations were critically appraised. In kidney transplantation, belatacept-based immunosuppression provides superior renal function and improved metabolic profiles compared with calcineurin inhibitors (CNIs), though with higher early acute rejection rates. In liver, heart, and lung transplantation, evidence remains limited, with de novo use contraindicated in liver grafts due to excess mortality and rejection. Conversion from CNI to belatacept in selected patients improves renal outcomes without compromising graft survival. Safety considerations include a higher risk of post-transplant lymphoproliferative disorder (PTLD) in Epstein–Barr virus-negative recipients. Belatacept represents a paradigm shift in transplant immunology by targeting upstream T-cell activation. While currently approved only for kidney transplantation, ongoing studies in thoracic and hepatic grafts may expand its therapeutic role. Personalized patient selection, combination regimens mitigating rejection risk, and real-world cost-effectiveness analyses will define its place in future precision immunosuppression strategies.

## 1. Introduction

The introduction of belatacept into clinical practice has reshaped the debate on long-term immunosuppressive strategies in solid organ transplantation. For decades, calcineurin inhibitors (CNIs)—primarily cyclosporine and tacrolimus—have formed the backbone of maintenance immunosuppression [1,2]. By selectively inhibiting a key enzyme in the T-cell signaling cascade, CNIs prevent transcription of interleukin-2 and other cytokines, thereby suppressing T-cell activation, proliferation, and differentiation [3]. Their widespread use has markedly reduced acute allograft rejection rates to <15% and improved 1-year renal graft survival to over 95% [4]. Despite their efficacy, chronic CNI exposure is associated with substantial nephrotoxicity [5], hypertension [6], dyslipidemia and neurotoxicity [7], new-onset diabetes after transplantation (NODAT) [8], and increased cardiovascular risk, all of which negatively affect long-term patient and graft survival [9]. CNIs therefore lack immunologic specificity, broadly affecting multiple pathways and contributing to cumulative toxicity [10]. Their narrow therapeutic window further necessitates individualized dose titration with therapeutic drug monitoring [11]. In addition to CNIs, antimetabolites such as mycophenolic acid remain widely used. Mycophenolate inhibits inosine monophosphate dehydrogenase (IMPDH), a rate-limiting enzyme in guanine nucleotide synthesis, selectively restricting T- and B-cell proliferation [12]. Mammalian target of rapamycin (mTOR) inhibitors—including sirolimus and everolimus—provide additional options for maintenance immunosuppression in kidney [13,14], heart [15], and liver transplantation [16]. Although better tolerated than CNIs, these agents are generally considered second-line therapies. Induction therapy with thymoglobulin, alemtuzumab, or basiliximab is often used to mitigate early rejection in the context of heightened immune activation [17]. While these regimens have improved outcomes, the need for better-tolerated, more selective immunosuppressive strategies remains high. Belatacept—a CTLA-4–Ig fusion protein engineered through two amino acid substitutions (L104E and A29Y) that markedly enhance affinity for CD80 and CD86—selectively blocks the CD28–CD80/86 costimulatory pathway essential for full T-cell activation [18]. Unlike abatacept, which was approved for rheumatoid and psoriatic arthritis [19,20] and later for prophylaxis of graft-versus-host disease [21], belatacept was specifically designed for solid organ transplantation. Its enhanced binding strength translated into potent in vivo immunoregulatory activity and demonstrated clinical promise in conditions requiring precise control of T-cell activation [22,23]. Acting upstream in the immune cascade, belatacept provides effective immunosuppression without nephrotoxicity and with a more favorable metabolic profile. However, concerns persist regarding early rejection risk, cost, and its limited role outside kidney transplantation [24]. In 2011, the EMA authorized belatacept in combination with corticosteroids and mycophenolic acid (MPA) for prophylaxis of graft rejection in adult kidney transplant recipients [25]. Belatacept is administered at transplantation with induction therapy (typically basiliximab), MMF, and corticosteroids. Corticosteroid tapering requires caution, particularly in high-risk patients with 4–6 HLA mismatches. Belatacept may also replace CNIs when they are poorly tolerated or ineffective. Conversion typically occurs ≥6 months post-transplant, using a 6 mg/kg schedule every 2 weeks for 8 weeks, then every 4 weeks, with CNIs maintained at reduced doses during the transition. This review summarizes core clinical trials supporting belatacept approval in kidney transplantation and examines additional evidence regarding its potential extension to heart, lung, liver, and pancreas transplantation. Moreover, a brief pharmacoeconomic analysis evaluates whether belatacept may offer advantages in clinical outcomes and healthcare resource utilization.

## 2. Methods

A comprehensive literature search was conducted to identify and critically appraise the available evidence on the use of belatacept in solid organ transplantation. This work was designed as a narrative, non-systematic review, aiming to provide a clinically oriented overview of both pivotal and real-world data. Given the exploratory nature of the review, no formal systematic review protocol was applied. The research strategy involved searches across multiple electronic databases, including PubMed/MEDLINE, Embase, the Cochrane Library, and ClinicalTrials.gov. On PubMed/MEDLINE, searches were performed using Boolean operators (AND, OR) to combine free-text terms and, where applicable, Medical Subject Headings (MeSH). The search strategy included terms such as “belatacept” AND (“solid organ transplantation” OR “kidney transplantation” OR “liver transplantation”), combined with additional keywords including “calcineurin inhibitors”, “acute rejection”, “graft survival”, “safety”, and “immunosuppression”. Similar strategies, adapted to the specific syntax of each database, were applied for the other electronic sources. In addition, regulatory documentation and publicly available assessment reports were retrieved from the European Medicines Agency (EMA) database, with particular attention to pivotal registration trials and post-authorization evidence. The search covered studies published from January 2005 to March 2025, encompassing early clinical development, registration studies, and contemporary clinical experience. Retrieved records were screened for relevance based on their consistency with the objectives of the review. Eligible publications included pivotal registration studies, randomized controlled trials (RCTs), prospective and retrospective cohort studies, observational studies, and, when clinically relevant, case reports and case series. Both interventional studies and real-world evidence were considered in order to capture the full spectrum of efficacy, safety, and practical implementation of belatacept-based immunosuppressive regimens. Reference lists of key articles were also manually screened to identify additional pertinent studies. Due to the narrative design of the review, no formal risk-of-bias assessment or meta-analytic approach was undertaken; however, data interpretation was based on a critical and balanced appraisal of the available evidence, taking into account study design, population characteristics, and potential sources of bias.

## 3. Belatacept in Organ Transplants

### 3.1. Belatacept in Kidney Transplantation

Belatacept has been most extensively studied in kidney transplantation, where it is currently approved for the prophylaxis of graft rejection in adults in combination with mycophenolic acid (MPA) and corticosteroids (Table 1). The pivotal BENEFIT and BENEFIT-EXT trials compared belatacept with cyclosporine in recipients of kidneys from standard-criteria donors (SCD) and extended-criteria donors (ECD), respectively [26,27,28,29,30]. Both consistently demonstrated superior renal function in belatacept-treated patients, with significantly higher eGFR at 1, 5, and 7 years post-transplant. In the BENEFIT phase III trial, a randomized, active-controlled study in adult recipients of SCD or living-donor kidneys, high patient and graft survival rates and improved renal function were maintained through 36 months [31]. Survival with a functioning graft at year 3 was 92% for both the more intensive (MI) and less intensive (LI) belatacept regimens, compared with 89% for cyclosporine. Improvements in renal endpoints—including reduced progression to advanced renal dysfunction—were primarily driven by higher eGFR in the belatacept groups. Acute rejection was more frequent with belatacept, occurring mainly within the first two years, and rarely recurring thereafter. BENEFIT-EXT, a phase III, multicenter, randomized trial in ECD recipients, reported similar patient and graft survival across treatment arms [32]. Acute rejection rates were comparable between groups, unlike in BENEFIT. Although ECD recipients exhibited lower mean GFR overall, renal function improved significantly with belatacept, with an expanding difference of approximately 11 mL/min at 36 months [32,33]. These findings suggest that belatacept may prolong graft longevity even in higher-risk ECD populations, who typically exhibit a twofold higher graft failure risk relative to younger-donor recipients [34]. Two extension analyses evaluated 84-month outcomes following the original 60-month follow-up [35]. In BENEFIT, belatacept reduced the risk of death or graft loss at 7 years by 43% compared with cyclosporine [36]. Hazard ratios (HRs) for death or graft loss were 0.52 and 0.48 at 60 months for MI and LI regimens, respectively, and 0.57 for both regimens at 84 months. These differences were statistically and clinically significant. BENEFIT-EXT subgroup analyses showed comparable death and graft loss rates between belatacept and cyclosporine, despite the overall higher-risk profile [37]. Seven-year outcomes stratified by donor subtype (UNOS ECD, DCD, CIT) revealed heterogeneous rejection patterns: cumulative acute rejection was lower in cyclosporine-treated UNOS ECD recipients but higher in the DCD and CIT cohorts. Nevertheless, renal function improvements remained greater with belatacept across subgroups.

Although belatacept approval is based on comparisons with cyclosporine, tacrolimus has long been the modern standard of care [38]. A retrospective observational cohort comparing belatacept and tacrolimus—alone or in combination—showed higher 1-year acute rejection risk with belatacept monotherapy, especially in patients receiving non-lymphocyte-depleting induction (HR = 2.65), but no difference in mortality [39]. Renal function at 1 year was superior in both belatacept monotherapy and combination groups versus tacrolimus alone. Because metabolic complications remain a concern, short-term tacrolimus co-administration during the first post-transplant year and induction therapy may reduce early rejection risk. Belatacept also offers a therapeutic alternative for patients initially maintained on CNIs who later develop toxicity (Table 2).

This effect is more pronounced in specific subpopulations, such as recipients of kidneys from living donors or standard criteria deceased donors, and in high-risk recipients (extended criteria donors, DCD, terminal creatinine ≥ 1.5 mg/dL and/or CIT ≥ 24 h), with an increase in eGFR at 12 months (60 vs. 54 mL/min/1.73 m^2^) [39]. Similar trends have been observed in retrospective studies on recipients of kidneys from deceased donors with pre-existing histopathological alterations, such as glomerulosclerosis, arteriosclerosis, and fibrosis, although statistical significance was not reached due to small sample sizes [40]. Another retrospective study on recipients from donors with acute kidney injury (AKI), stratified by stages I–III, showed significantly better graft function at 6 months in patients treated with belatacept compared to tacrolimus, with clinically modest differences that were no longer significant at 12 months [41]. However, increased heterogeneity due to sample expansion may mask this effect, as observed in another retrospective study where no significant difference was found between belatacept and tacrolimus regarding the composite endpoint of death, graft loss, or eGFR < 45 mL/min/1.73 m^2^ at 12 months. Nonetheless, other notable aspects emerge, such as significantly lower CNI-induced neurotoxicity compared to belatacept [42]. Indeed, from a safety perspective, belatacept shows a more favorable profile than CNIs, with lower incidence of neurotoxicity, hypertension, hypercholesterolemia, and chronic nephrotoxicity, which are clinically relevant in long-term graft management [42]. However, belatacept requires monthly or bi-monthly intravenous administrations, which may pose greater logistical challenges compared to tacrolimus, available orally and allowing easier home self-administration. Other practical considerations include the need for more frequent monitoring in high-risk patients and the absence of long-term experience relative to tacrolimus.

In an exploratory phase II trial (NCT00402168), stable kidney transplant recipients ≥ 6 months post-transplant were randomized to either conversion to belatacept or continuation of CNI therapy [43]. Conversion resulted in improved renal function, high patient and graft survival, and a low incidence of acute rejection, despite inherent conversion risks and the avoidance of CNI-associated toxicities. A decade later, conversion was reassessed in a phase 3b prospective trial (NCT01820572) in recipients 6–60 months post-transplant [44]. The primary endpoint—survival with a functioning graft at 24 months—was achieved in 98% of the belatacept group versus 97% of the CNI group. Renal function again favored belatacept. Although earlier conversion may yield greater benefit by limiting CNI toxicity, current approval supports conversion only after 6 months [45]. Biopsy-proven acute rejection (BPAR) was slightly higher in the belatacept arm, occurring within the first 6–7 months and responding to therapy. A novel combination regimen was evaluated in a randomized phase II trial (NCT02137239) comparing belatacept plus everolimus with tacrolimus plus MMF following rATG induction and early corticosteroid withdrawal [46]. Acute rejection rates through 2 years were similar across groups, with no deaths or graft losses attributable to rejection. Additional regimens were tested in an open-label randomized phase II study (NCT00455013), comparing belatacept + MMF, belatacept + sirolimus, and tacrolimus + MMF following rATG induction without steroids [47]. Acute rejection by month 6 was low but slightly higher in the belatacept + MMF arm (12%) versus belatacept + sirolimus (4%) or tacrolimus + MMF (3%). Nevertheless, rejection rates compared favorably to other CNI- or steroid-free protocols (12% vs. 36%) [48]. Renal function at 12 months was superior in belatacept-based groups, supporting the feasibility of steroid- and in some cases CNI-free regimens. However, small sample size limited power.

### 3.2. Belatacept in Liver Transplantation

The promising outcomes observed with belatacept in kidney transplantation have not been replicated in liver transplantation. A randomized phase II trial (NCT00555321) evaluating de novo belatacept in liver transplant recipients was terminated early due to unacceptably high rates of acute rejection, graft loss, and mortality compared with tacrolimus-based therapy [49]. By month 6, belatacept-treated groups had already reached significantly higher rates of the trial’s composite adverse outcomes. At 1 year, mortality and graft loss remained increased—primarily due to sepsis, multiorgan failure, or gastrointestinal bleeding—despite improvements in GFR similar to those previously observed in kidney transplantation. Patients undergoing liver transplantation often represent a clinically complex population, characterized by severe comorbidities and advanced baseline disease, factors that may negatively impact post-transplant outcomes independently of belatacept use. In this context, non-immunological variables, such as the severity of liver disease at the time of transplant, the extent of surgical trauma, and significant perioperative hemodynamic and fluid balance alterations, may have substantially contributed, albeit indirectly, to the unfavorable clinical outcomes observed in the study. In addition, the included patients were often already immunocompromised prior to liver transplantation, which may have further modulated responses to the evaluated immunosuppressive regimens. The study analyzed various therapeutic protocols, mainly based on concomitant immunosuppression with induction or maintenance therapies [49]. However, patient allocation to different regimens was performed in an open-label, non-randomized design, representing a significant methodological limitation potentially associated with selection and performance bias, which may have influenced the interpretation of results. The belatacept doses employed, higher than those used in kidney transplantation, did not show a clear correlation with mortality or graft loss, even in the absence of TDM, representing another limitation. The higher rates of acute rejection in the belatacept groups suggest possible under-immunosuppression or immunomodulatory mechanisms not yet clarified in liver transplantation. Intensification of anti-rejection treatment may have increased the risk of infections; however, this association could not be statistically demonstrated due to the limited sample size.

The pathophysiological reasons for poor outcomes remain incompletely understood. Differences in allograft immunobiology—particularly the balance between T cell–mediated rejection (TCMR) and antibody-mediated rejection (AMR)—likely contribute [50]. In kidney transplantation, TCMR predominates and is responsive to CD28–CD80/86 pathway blockade, whereas in liver transplantation, AMR may progress rapidly to chronic rejection [51]. Biopsies from renal recipients experiencing TCMR reveal no major differences in effector T-cell responses between belatacept and CNI treatment, suggesting similar injury mechanisms across regimens [52]. The poor outcomes seen in liver transplantation may stem from B-cell–mediated pathways, including alloantibody production and pro-inflammatory cytokine secretion [53]. Endothelial injury, altered antigen expression, and shifts in the antibody repertoire may further amplify rejection [54]. While AMR is uncommon in liver transplantation due to intrinsic immunotolerance, when it occurs it is often severe. Another potential explanation involves belatacept’s selective inhibition of CD28+ T helper cells. CNIs, by contrast, exert broader immunosuppressive effects, including suppression of CD28− T helper subsets, thereby reducing alloantibody generation more effectively [55]. CD28− T-cell populations expand with age and chronic inflammation due to increased levels of TNF, IL-2, and IL-15, which drive loss of CD28 expression [56,57]. Induction with basiliximab—blocking the IL-2 receptor—followed by belatacept may explain slightly better survival in select studies compared with MI and LI belatacept monotherapy regimens. The divergent clinical outcomes observed between kidney and liver transplantation may, at least in part, be explained by intrinsic immunobiological differences between the two allografts, particularly regarding the degree of tolerance, the role of alloantibodies, and the contribution of antibody-mediated rejection (AMR). Among established evidence, the relative immunotolerance of the liver is greater than that of the kidney, allowing the graft to maintain function even in the presence of circulating alloantibodies. Phenomena such as accommodation suggest that in certain circumstances the allograft may function despite the presence of donor-specific antibodies (DSA), due to cytoprotective and endothelial adaptation mechanisms; however, this favors graft function but does not explain the higher incidence of acute rejection. An important aspect concerns the differential role of AMR in the two organs: in kidney transplantation, AMR is a frequent cause of long-term graft loss, whereas in the liver it is less common, likely due to the organ’s high regenerative and immunomodulatory capacity. However, when AMR occurs in the liver, it is often severe and rapidly progressive, leading to chronic graft dysfunction. Furthermore, B cells play a complex role, including antibody production, antigen presentation, cytokine secretion, and regulation of immune responses. In the kidney, the presence of B cells associated with operational tolerance suggests that specific regulatory B cell (B_reg_) subsets may contribute to long-term graft survival [58]. Hypotheses underlying the differential acute rejection between kidney and liver transplants may explain these disparities in immunosuppressive response. One possible explanation is that the liver, although more tolerant, may be particularly vulnerable when the balance between accommodation and humoral response is disrupted. In this context, inadequately controlled antibody responses could rapidly induce endothelial injury, complement activation, and graft loss, whereas AMR progression in the kidney tends to be more gradual. It is plausible that in liver transplantation, B cell responses are skewed toward pro-inflammatory alloantibody production, whereas in kidney transplantation, regulatory B cell phenotypes may be more readily induced. This difference could influence the capacity to develop operational tolerance or maintain stable accommodation over time. Differences in lymphoid architecture, antigen presentation, and the inflammatory microenvironment between liver and kidney may also substantially affect B cell contributions to T-dependent responses, with divergent consequences for clinical outcomes [58].

Based on current evidence, regulatory agencies such as the EMA contraindicate belatacept for de novo use in liver transplantation due to high rejection and graft loss rates. More robust clinical data are required before reconsideration of approval.

Despite these limitations, belatacept has occasionally been used as “rescue” therapy in patients who develop severe CNI-induced nephrotoxicity after liver transplantation. In such cases, conversion from CNIs to belatacept has been associated with improved renal outcomes without substantial increases in rejection risk, although evidence remains anecdotal and limited to small case series [59,60,61]. Thus, while unsuitable as first-line therapy, belatacept may retain a niche role as salvage immunosuppression in carefully selected liver transplant recipients.

### 3.3. Belatacept in Heart and Lung Transplantation

Evidence for belatacept in thoracic organ transplantation remains limited. In heart transplantation, standard first-line pharmacological therapy includes a calcineurin inhibitor (CNI) combined with mycophenolate mofetil (MMF) and corticosteroids. As a second-line option, everolimus with early cyclosporine withdrawal may reduce progression of cardiac allograft vasculopathy (CAV) at 12 and 36 months compared with conventional CNI-based therapy [62,63]. Long-term CNI exposure, however, contributes to substantial nephrotoxicity and cardiometabolic complications, prompting interest in alternative strategies [63]. A small number of case reports and case series describe the use of belatacept in CNI-intolerant heart or lung transplant recipients, mainly to preserve renal function or treat progressive CNI toxicity [64,65]. One early report described a 26-year-old heart transplant recipient with postpartum cardiomyopathy who had become non-adherent to a tacrolimus-based regimen; belatacept (10 mg/kg) successfully prevented both cellular and antibody-mediated rejection, with biopsies remaining grade 0 [64]. Another case involved a 27-year-old woman who underwent heart transplantation followed later by kidney transplantation; a regimen including belatacept, tacrolimus, MMF, and prednisone stabilized both grafts, consistent with BENEFIT trial observations [65]. Although these reports suggest acceptable graft outcomes and renal stabilization under belatacept-based regimens, they lack statistical power and include heterogeneous patient populations. A retrospective cohort of heart transplant recipients—including highly sensitized patients, individuals with donor-specific antibodies (DSA), prior antibody-mediated rejection (AMR), or impaired renal function—also demonstrated preserved renal function, low rejection rates, and maintained allograft function, though sample size and immunologic diversity limit interpretation [66]. An observational study of 40 heart transplant patients similarly reported improved renal function with a favorable safety profile [67]. The largest cohort to date showed significant renal benefits with belatacept, with 2-year survival of 98.5% [68], while rejection rates and freedom from AMR at 3 years did not differ significantly from controls. Notably, belatacept-treated patients exhibited slower progression of pre-existing CAV. Regulatory agencies do not currently approve belatacept for heart or lung transplantation due to insufficient robust evidence. Ongoing studies may clarify its role. A phase II pilot trial (NCT04477629) is evaluating de novo belatacept in heart transplantation with MMF, corticosteroids, and gradual tacrolimus reduction, primarily to assess safety [69]. Another prospective, multicenter, open-label phase II study (NCT06478017) is investigating phased tacrolimus withdrawal with belatacept over 9 months as a strategy to prevent a composite endpoint including acute cellular rejection, hemodynamic compromise, retransplantation, or death at 18 months [70]. In lung transplantation, a phase II pilot study evaluated belatacept-based immunosuppression combined initially with tacrolimus and prednisone, followed by MMF (NCT03388008). Despite no need for retransplantation in either arm, higher mortality (38%) and increased susceptibility to infection were observed in the belatacept group [71]. Another pilot study assessed belatacept as a bone marrow-sparing strategy in lung transplant recipients with idiopathic pulmonary fibrosis (IPF) and short telomeres [72]. Belatacept was used as a bridge to everolimus to avoid early airway dehiscence associated with mTOR inhibitors. Acute cellular rejection was low (11%), but prolonged belatacept exposure correlated with EBV viremia and post-transplant lymphoproliferative disorder (PTLD). Most cases occurred in patients receiving long-term belatacept (8–20 months), suggesting exposure duration as a critical risk factor, particularly in patients predisposed to herpesvirus infections and PTLD due to short telomeres. Thoracic transplant recipients face elevated baseline risks of infection and malignancy, amplifying belatacept’s safety concerns, particularly regarding PTLD. While belatacept may be an option for patients with cytopenias or intolerance to cell cycle inhibitors, prolonged use carries significant risk. A single-center retrospective chart review of lung transplant recipients converted to belatacept-based immunosuppression found no significant differences in acute cellular rejection before versus after conversion (*p* = 0.17) [73]. eGFR improved significantly post-conversion, while infection incidence and blood pressure remained stable. Two patients developed chronic lung allograft dysfunction (CLAD), and 7 of 11 patients were alive at last follow-up. Additional small series indicate belatacept may temporarily allow safe reduction in conventional immunosuppression, stabilizing or improving renal function in patients with acute or chronic renal impairment [74]. Given current evidence, belatacept is not approved for heart or lung transplantation and should be used only in clinical trials or highly selected clinical scenarios requiring rescue therapy. Nevertheless, its potential role in CNI-sparing regimens or as part of combination strategies remains a subject of ongoing research.

## 4. Safety of Belatacept

### 4.1. Common Side Effects Versus CNIs

The safety profile of belatacept requires careful evaluation. In the BENEFIT trial, which compared belatacept-based immunosuppression with cyclosporine, advantages in patient/graft survival and renal function were accompanied by key safety considerations [36]. The most frequently reported adverse effects at 36 months included flatulence, fatigue, lack of energy, and restlessness or nervousness. Some symptoms meaningfully affected quality-of-life metrics, particularly dyspnea, voice alteration, muscle weakness, joint pain, depressed mood, anxiety, and gastrointestinal disturbances. Patients treated with belatacept achieved significantly better physical component scores (PCS). Several side effects—gingival swelling, peripheral edema, tremor, hirsutism, and oily skin—occurred less frequently with belatacept than with CNIs, whereas dry skin was slightly more common. In BENEFIT-EXT, adverse events such as anemia, graft dysfunction, urinary tract infection, diarrhea, constipation, peripheral edema, pyrexia, and hypertension occurred at similar rates between treatment groups, while nausea, leukopenia, and vomiting were more frequent with belatacept [32]. Serious adverse events, including cytomegalovirus (CMV) and urinary tract infections, were comparable. Belatacept also demonstrated a lower risk of cardiovascular and metabolic complications versus cyclosporine, including reduced blood pressure, improved lipid profiles, and lower incidence of new-onset diabetes after transplantation (NODAT), although other safety concerns emerged [26].

### 4.2. Serious Risks (e.g., PTLD, Infections), Stratified by EBV Status and Organ Type

The primary risk associated with belatacept is post-transplant lymphoproliferative disorder (PTLD), particularly involving the central nervous system, with cases occurring as early as the first year [32,36,75]. PTLD risk is highest among Epstein–Barr virus (EBV)-seronegative recipients, leading to a strict contraindication for belatacept use in this population [32,36,76]. The underlying mechanism relates to profound T-cell suppression, which impairs EBV-specific immune surveillance and permits uncontrolled proliferation of EBV-infected B cells [77]. EBV-mediated immune evasion and viral oncogene-driven inhibition of apoptotic pathways further contribute to PTLD development [78,79]. Consequently, pre-transplant EBV serological screening is mandatory for belatacept therapy [80]. PTLD occurs more often in heart and lung transplant recipients than in kidney or liver recipients, likely reflecting more intense immunosuppression, though further investigation is needed [81]. Infectious complications—including CMV, BK virus, pneumonia, pyelonephritis, and sepsis—may occur at similar or slightly higher frequencies with belatacept compared with CNIs. These patterns mirror those observed in other immunosuppressive regimens [75]. Late CMV reactivation may stem from progressive loss of CMV-specific T-cell responses under continuous T-cell suppression [82]. Belatacept combined with mTOR inhibitors has shown potential to restore CMV-specific αβ and γδ T-cell responses, supporting individualized immune monitoring in high-risk CMV-seropositive patients [83]. CMV disease may also predict PTLD after primary EBV infection in liver transplant recipients, especially when EBV-negative recipients receive EBV-positive grafts [84]. Hepatitis C virus (HCV) infection has been implicated as an additional PTLD risk factor in the presence of EBV [85]. Long-term malignancy risk remains an open question. Belatacept induces prolonged immunosuppression, which is not rapidly reversible in the event of acute infections, with relevant clinical implications for the management of transplant recipients. During the SARS-CoV-2 pandemic, kidney transplant recipients treated with belatacept showed a non-negligible risk of progression to severe COVID-19, particularly in symptomatic patients, highlighting the need for careful clinical monitoring [86]. Therefore, the management of these patients requires close surveillance and case-by-case evaluation of potential adjustments to the immunosuppressive regimen, as well as prompt hospital care when needed. Humoral immune responses to mRNA SARS-CoV-2 vaccination in patients treated with belatacept were also significantly lower compared with those observed in patients receiving tacrolimus. In a specific cohort, only 5.7% of kidney transplant recipients on belatacept developed detectable antibodies after the vaccination cycle, a phenomenon attributed to the concomitant administration of the vaccine with the drug infusion, which may reduce immunogenicity [87]. In a subsequent study, when vaccination was scheduled 21 days after belatacept infusion, a significant improvement in antibody response was observed, with 17% seropositivity after the second dose, suggesting that the timing of drug and vaccine administration can substantially influence the effectiveness of the induced immune response [88]. The ability to develop effective antibody responses is also modulated by individual clinical factors: advanced age, diabetes mellitus, impaired renal function (low eGFR), concomitant use of MPA or MMF, high-dose corticosteroids, and combined immunosuppressive regimens are associated with a higher risk of negative antibody response, whereas the use of mTOR inhibitors appears to have a protective effect [89].

### 4.3. Long-Term Risks (e.g., Malignancy, Metabolic Effects)

Safety comparisons with tacrolimus show a similar pattern. In the observational study cited earlier [39], belatacept displayed better metabolic tolerability, including significantly lower NODAT incidence (1.7% with belatacept + tacrolimus, 2.2% with belatacept alone, vs. 3.8% with tacrolimus; *p* = 0.01). PTLD and de novo malignancies occurred at similar rates across groups. Although improved graft function and metabolic profiles favor belatacept monotherapy, short-term tacrolimus during the first year remains advisable in many patients. Additional safety data from a study comparing belatacept + MMF or belatacept + sirolimus with tacrolimus + MMF showed similar incidences of common adverse events—including anemia, pyrexia, leukopenia, diarrhea, and constipation—across arms [47]. Aphthous stomatitis and proteinuria occurred only in belatacept regimens, while tremor and wound dehiscence were more frequent with tacrolimus. Hydronephrosis and pyrexia were the most common serious adverse events. Overall, belatacept’s safety profile aligns with findings from de novo renal transplantation trials, showing favorable tolerability and improved adherence due to once-monthly dosing. While infusion requirements pose logistical challenges and add costs, scheduled administration enables close monitoring and may enhance compliance relative to self-administered oral CNI regimens. The need for therapeutic drug monitoring (TDM) of CNIs primarily represents a managerial and economic requirement rather than a true clinical limitation, as it allows individualized dose titration based on pharmacological exposure and the patient’s immunological risk. However, TDM of CNIs is associated with direct costs related to laboratory analyses for plasma drug level determination, as well as indirect costs for both patients and healthcare systems, including repeated hospital visits, blood sampling, and overall logistical burden. Conversely, a potential limitation of belatacept lies in the absence of a validated and routinely applicable TDM strategy in clinical practice. The lack of tools for monitoring pharmacological exposure currently prevents individualized dose optimization according to immunological risk profile, clinical evolution, or immune response at the individual patient level. Although belatacept is characterized by more predictable pharmacokinetics and does not require routine monitoring according to its Summary of Product Characteristics, the inability to personalize therapy through TDM-based strategies represents a clinically relevant limitation when compared with CNI-based immunosuppressive regimens.

### 4.4. Belatacept in Hepatocellular Carcinoma Liver Transplant Recipients

In patients undergoing liver transplantation for hepatocellular carcinoma (HCC), the goal of immunosuppression is not limited to preventing rejection, but requires maintaining a delicate dynamic balance between controlling alloimmune responses and preserving antitumor immune surveillance. Excessive immunosuppression can promote tumor progression or recurrence, while insufficient immunosuppression increases the risk of acute and chronic graft rejection. Use of a belatacept-based immunosuppressive regimen is not recommended in patients concurrently receiving anti-CTLA-4 antibodies for tumor therapy, due to the intrinsic mechanisms of these agents. While CTLA-4 blockade enhances T-cell activity against tumors, belatacept, as a structural mimic of the extracellular domain of CTLA-4, blocks costimulation and suppresses T lymphocytes. This creates a potential conflict in the oncological management of HCC post-transplant, although combination immunotherapy approaches may represent a promising future strategy [90]. Tumor recurrence after transplantation is influenced not only by carcinoma type and immunosuppressive regimen, but also by intraoperative surgical factors. In this context, the “no-touch” hepatectomy technique, which limits manipulation of the neoplastic liver before vascular control, can reduce intraoperative dissemination of tumor cells and indirectly improve clinical outcomes [91]. Therefore, targeted surgical strategies and carefully balanced immunosuppressive regimens should be considered complementary components of integrated oncological management. In this perspective, the use of immunosuppressive agents with selective immunomodulatory profiles, such as belatacept, warrants critical evaluation within personalized approaches aimed at maintaining the balance between rejection prevention and tumor control. Belatacept use in this setting should be approached with caution and reserved for selected contexts, pending dedicated studies clarifying its impact on the equilibrium between immune tolerance and oncological control.

## 5. Pharmacoeconomic Considerations

The cost of belatacept is substantially higher than that of standard CNI-based immunosuppressive regimens. However, despite the higher drug acquisition costs and based on clinical data from the studies previously discussed, long-term savings may potentially emerge from the reduction in nephrotoxicity, cardiovascular complications, and dialysis dependence associated with chronic exposure to CNIs. Additional costs would include hospital expenses related to the probability of transitioning to specific health states such as acute rejection, graft loss, or death. Health-related quality of life (HRQoL) represents another relevant outcome in kidney transplantation. Data from the BENEFIT and BENEFIT-EXT studies showed improvements in the physical and mental components of the SF-36 questionnaire at 12, 24, and 36 months [92]. However, although the maximum improvement reached statistical significance (*p* = 0.01) for the physical component score (PCS) in the belatacept group compared to the cyclosporine group, the difference was only approximately 3 points on a 0–100 scale (46.4 vs. 43.6–6% difference). Better renal function was associated with higher PCS and mental component scores (MCS), whereas deterioration of renal function correlated with lower HRQoL. Although belatacept-treated patients reported fewer side-effect-related symptoms than cyclosporine-treated patients, differences did not reach statistical significance, despite a consistent trend favoring belatacept. A complementary cross-sectional survey evaluating patient experience with monthly belatacept infusions found high overall satisfaction, despite logistical burdens such as travel, infusion duration, and scheduling constraints [93]. Aligning infusions with laboratory visits may reduce indirect costs and inconvenience. The convenience of once-monthly dosing may also enhance adherence relative to daily oral CNI regimens. Nevertheless, kidney transplant recipients still experience higher symptom burden than the general population, reflecting multifactorial influences on HRQoL [94]. A post hoc longitudinal analysis from BENEFIT and BENEFIT-EXT further demonstrated that HRQoL trajectories strongly correlate with clinical outcomes [95]. Most patients showed sustained improvements in HRQoL post-transplant, yet several subgroups exhibited progressive decline over three years. Latent class analysis identified distinct trajectories across SF-36 subscales. General health followed a four-class model, with the largest class (43%) maintaining scores near the general population mean. Symptoms such as anxiety and muscle cramps at 12 months were significantly associated with lower HRQoL trajectories, while sleep disturbances and headaches were relevant at baseline. Persistent symptoms suggest the need for targeted interventions to improve long-term HRQoL. Despite these benefits, most healthcare systems restrict belatacept use to specific high-risk populations due to its higher cost. Annual pharmaceutical expenditures for CNI-based regimens remain substantially lower. For example, a 70 kg patient treated with immediate-release tacrolimus at 0.15 mg/kg/day represents an approximate annual cost of €3492.24, while cyclosporine therapy (10→6 mg/kg/day) costs approximately €1847.82 in the first post-transplant year [96,97]. By contrast, a 70 kg patient receiving belatacept requires six 10 mg/kg doses during the first three months (700 mg per infusion), followed by 6 mg/kg monthly (420 mg). This equates to 18 vials during the induction phase and 20 vials during maintenance in the first year, totaling €15,352.00 based on ex-factory pricing [98]. Conversion therapy initiated at month six lower costs to €6464.00 for the first year (16 vials required). Infusion-based therapy also imposes higher institutional costs. While CNI-based regimens can be managed through territorial healthcare services once therapeutic levels are stabilized, belatacept infusions must be administered in hospital settings, increasing pharmaceutical and logistical expenses. Indirect costs include patient travel, meals, and productivity losses due to 16 infusion visits in the first year, affecting both patients and caregivers. Nevertheless, the clinical benefits of belatacept—including improved renal function and reduced CNI-related toxicity—may offset long-term costs. Indeed, re-transplantation due to CNI-induced toxicity entails significant hospital costs, encompassing both the pre- and post-transplant phases, with expenses that may vary considerably across different countries. In Italy, a potential organ re-transplantation due to CNI-induced toxicity would lead to a significant increase in hospital costs, encompassing expenses related to both the pre- and post-transplant phases, and would also require the immediate availability of the organ in question [99]. In heart transplantation, where secondary kidney transplantation is common due to chronic CNI nephrotoxicity [100,101,102], belatacept may lower overall healthcare expenditure by preventing renal replacement therapy. Belatacept also reduces tacrolinemia monitoring frequency, lowering laboratory-related costs. Coordinated infusion scheduling may further reduce per-patient drug expenditure. Treating six patients on the same infusion day, for example, allows vial optimization such that six patients can be treated at the cost of five, provided close collaboration between clinical teams and hospital pharmacy services. Ultimately, cost-effectiveness will depend on broader therapeutic indications, pricing negotiations, long-term comparative outcomes, and real-world pharmacoeconomic data across healthcare systems. Currently, specific health economic analyses focused on cost-utility (cost per quality-adjusted life year, QALY) using appropriate decision models are lacking. Therefore, such analyses should be conducted before belatacept can be considered as a first-line therapy in kidney transplantation.

## 6. Conclusions and Future Directions

Over the years, the choice of an appropriate immunosuppressive regimen for induction and maintenance therapy following solid organ transplantation has been driven by the need to use drugs that are both effective and associated with fewer adverse effects, thereby reducing patient morbidity and mortality. In this context, the need for personalized immunosuppressive strategies arises from the fact that an optimal standard therapy has not yet been established [100,101]. Patient genotyping represents an increasingly relevant consideration, as it could help reduce inter-individual variability in response to CNIs due to genetic polymorphisms in cytochromes [102]. Variants such as CYP3A5 expression in liver donors or the 2677T/A allele in kidney transplant recipients significantly affect the pharmacokinetics of tacrolimus [103]. Although genetic stratification may improve the appropriateness of use and the safety of CNIs across different types of transplantation, its impact on overall clinical outcomes remains uncertain, since modulation of pharmacokinetics alone may not be sufficient to guarantee therapeutic efficacy. Belatacept represents a paradigm shift in transplant immunology by targeting upstream T-cell activation rather than downstream cytokine pathways. Its introduction has significantly reshaped kidney transplantation, demonstrating superior renal function and favorable metabolic outcomes compared with calcineurin inhibitors (CNIs). The major challenge now lies in expanding its use to other solid organs—including the heart, lung, and liver. This possibility is clinically relevant, as many thoracic and hepatic transplant recipients ultimately develop progressive CNI-induced nephrotoxicity that may require renal replacement therapy or secondary kidney transplantation [104,105,106]. By preventing chronic CNI toxicity, belatacept could help avoid the need for additional organ transplantation, with substantial implications for patient outcomes and healthcare resource utilization. Current evidence from controlled trials and real-world studies supports potential use of belatacept as a first-line option in selected high-risk populations, especially those predisposed to chronic kidney injury. Belatacept also lacks many CNI-related cardiovascular, metabolic, and neurologic toxicities, which are major determinants of long-term survival in cardiothoracic transplantation. Moreover, belatacept has been associated with lower rates of new-onset diabetes after transplantation (NODAT), improved blood pressure control, and more favorable lipid profiles—effects especially valuable in patients with elevated cardiovascular risk. However, in heart, lung, and liver transplantation, evidence regarding patient and graft survival remains limited. As a result, belatacept cannot currently be considered standard of care in these settings and should generally be reserved for rescue therapy or inclusion in clinical trials. Key challenges include early rejection risk, higher drug cost, infusion requirements, and contraindication in EBV-seronegative recipients due to the increased risk of post-transplant lymphoproliferative disorder (PTLD). Although rejection episodes have generally been manageable with standard therapies, these safety concerns continue to restrict broader adoption. Ethical considerations also arise during conversion from stable CNI therapy. Patients may fear rejection or instability associated with switching from a familiar regimen. Clinicians must therefore balance long-term risks of CNI toxicity—including nephrotoxicity, cardiovascular disease, and metabolic complications—against the acute risks associated with conversion, ensuring informed and shared decision making. Patient support through telemedicine may represent a useful strategy to prevent hospitalizations or to manage critical situations, as has been proposed in other clinical settings [107]. Despite logistical challenges, monthly intravenous administration offers advantages such as structured monitoring and potentially improved adherence relative to self-administered oral CNIs. Although more costly than CNIs, the improved renal and metabolic outcomes associated with belatacept may translate into long-term cost savings in selected populations. Belatacept also appears to carry a lower risk of advanced cutaneous squamous cell carcinoma (CSCC) and may reduce the incidence of non-melanoma skin cancers (NMSC), an important consideration for fair-skinned individuals and patients with prior dermatologic malignancies [108,109,110].

Going forward, research priorities include:

- Identifying biomarkers to guide patient selection and predict belatacept responsiveness;
- Refining combination regimens to mitigate early acute rejection risk;
- Conducting large randomized trials in non-renal organ transplantation;
- Clarifying long-term safety, particularly the risk of malignancy and infection;
- Generating real-world pharmacoeconomic evidence across diverse healthcare systems.

Careful patient selection is essential to optimize the benefit–risk profile of belatacept-based regimens. The ideal candidate for de novo kidney transplantation is an EBV-seropositive recipient with low-to-intermediate immunological risk, preferably without preformed donor-specific antibodies (DSA), receiving depleting induction therapy, and not undergoing CTLA-4 inhibitor treatment for oncology indications. The greatest benefits in preserving renal function have been observed in recipients of kidneys from living donors or standard-criteria deceased donors, particularly in high-risk graft scenarios (extended criteria donors, DCD, terminal creatinine ≥ 1.5 mg/dL, and/or CIT ≥ 24 h). Belatacept may also be advantageous for patients with metabolic or cardiovascular comorbidities, due to its favorable profile compared with calcineurin inhibitors (CNIs). Conversion to belatacept is particularly indicated in EBV-seropositive patients who develop CNI-induced nephrotoxicity, other significant adverse events (neurotoxicity, hypertension, or metabolic disturbances), or progressive renal function decline, especially when performed early after transplantation. Absolute contraindications include EBV seronegativity and a history of post-transplant lymphoproliferative disease, while caution is warranted in patients with high immunological risk, persistent DSA, or logistical challenges that prevent regular intravenous administration and adequate follow-up.

Ultimately, belatacept represents the next frontier in precision immunosuppression—offering the potential to minimize toxicity while maximizing long-term graft and patient survival. As knowledge of transplant immunobiology advances, belatacept and next-generation costimulation blockers will likely play increasingly central roles in shaping the future landscape of solid organ transplantation.

## Figures and Tables

**Table 1:** Data from major Belatacept clinical trials.

Trial (NCT)/Phase	Organ/Population	Comparator/Regimen	Primary Outcomes	Acute Rejection	Renal Function/Graft Outcomes	Survival/Safety	Notes
BENEFIT (NCT00256750)—Phase III	Kidney—living or standard-criteria deceased donor	Belatacept (MI & LI) vs. Cyclosporine A	Patient & graft survival, measured GFR, acute rejection	CsA 7.2%; Bel-LI 17.2%; Bel-MI 21.9%	Higher GFR vs. CsA at 12 months	Non-inferior patient/graft survival	Landmark pivotal trial; better renal function, more AR events
BENEFIT-EXT (NCT00114777)—Phase III	Kidney—extended-criteria donor	Belatacept (MI & LI) vs. CsA	Survival with graft, GFR thresholds, acute rejection	CsA 14.1%; Bel-LI 17.7%; Bel-MI 17.4%	Improved renal function vs. CsA	Comparable survival	Extended-criteria donors; renal benefit despite higher AR
IM103-010 (NCT00402168)—Phase II/III	Kidney—stable recipients (conversion)	Switch to belatacept vs. continue CsA/TAC	Change in GFR, AR incidence, safety	Acceptable AR; improved renal function	Improved eGFR vs. CNI continuation	Comparable safety	Supports CNI-sparing conversion approach
NCT01820572—Phase II/III	Kidney—stable post-transplant (6–60 months)	Conversion to Belatacept vs. CNI	% survival with functional graft, renal function	Reported AR within expected range	Improved renal function post-conversion	Comparable survival	Sponsor: BMS; ongoing long-term follow-up
NCT04477629—Phase II (open-label)	Heart—de novo adult transplant recipients	Belatacept-based (de novo)	Safety assessment, feasibility	Ongoing	To be determined	Preliminary safety focus	Exploratory CNI-sparing approach; results pending
NCT06478017—Phase II	Heart—gradual TAC withdrawal	Belatacept + TAC taper vs. TAC standard	Safety, rejection, graft function	Ongoing	Pending	Preliminary safety signals only	Mitigates AR risk via delayed CNI withdrawal
NCT03388008—Phase II (pilot)	Lung— de novo transplant	Belatacept-based vs. standard CNI	Feasibility, acute rejection, infection	Data not yet complete	Renal protection potential	Limited pilot sample	Small-scale proof-of-concept; awaiting full results
NCT03781414/CCFZ533A2202—Phase II	Liver—adult recipients	Belatacept vs. Tacrolimus-based	Graft survival, safety	High AR and graft loss in some arms	Unfavorable renal/graft outcomes	Higher mortality, PTLD, PML cases	Regulatory warning: belatacept not recommended in liver Tx

**Table 2:** Efficacy, safety, and practical logistics of belatacept-based regimens versus tacrolimus-based regimens.

Aspect	Belatacept	Tacrolimus
Acute rejection at 1 year	Higher risk in monotherapy without depleting induction	Lower risk in all settings
Renal function (eGFR)	Better, especially in high-risk subgroups	Lower compared to belatacept
Safety	Lower nephrotoxicity, neurotoxicity, and hypertension	Higher risk of nephrotoxicity and neurotoxicity
Administration	Intravenous, monthly/bi-monthly	Oral, daily
Logistics	Requires hospital access and monitoring	Self-managed at home with standard monitoring

## Data Availability

No new data were created or analyzed in this study. Data sharing is not applicable to this article.
